# Fulvic acid alleviates cadmium-induced root growth inhibition by regulating antioxidant enzyme activity and carbon–nitrogen metabolism in apple seedlings

**DOI:** 10.3389/fpls.2024.1370637

**Published:** 2024-04-02

**Authors:** Bo Yu, Xiaomin Xue, Peixian Nie, Ninglin Lu, Laiping Wang

**Affiliations:** ^1^ Shandong Institute of Pomology, Shandong Key Laboratory of Fruit Biotechnology Breeding, Taian, China; ^2^ College of Horticulture, Shenyang Agricultural University, Shenyang, China

**Keywords:** apple, cadmium, fulvic acid, antioxidant capacity, photosynthetic performance, C and N metabolism

## Abstract

**Introduction:**

Substantial previous studies have reported that fulvic acid (FA) application plays an important role in Chinese agricultural production. However, little is known about the mechanisms for using FA to increase apple trees resistance to Cd toxicity. In order to clarify the mechanism underlying FA alleviation in Cd-induced growth inhibition in apple seedlings.

**Methods:**

Herein, we treated M9T337 seedlings to either 0 or 30 µM/L Cd together with 0 or 0.2 g/L FA and analyzed the root growth, antioxidant enzyme activities, carbon (C) assimilation, nitrogen (N) metabolism, and C and N transport.

**Results:**

The results presented that, compared with CK (without Cd addition or FA spraying application), Cd poisoning significantly inhibited the root growth of apple seedlings. However, this Cd-induced root growth inhibition was significantly alleviated by FA spraying relative to the Cd treatment (Cd addition alone). On the one hand, the mitigation of inhibition effects was due to the reduced oxidative damage by enhancing antioxdiant enzyme (SOD, POD, and CAT) activities in leaves and roots. On the other hand, this growth advantage demonstrated compared to the Cd treatment was found to be associated with the strengthen of photosynthetic performance and the elevation of C and N metabolism enzymes activities. Meanwhile, we also found that under Cd stress condition, the distribution of C and N nutrients in apple seedlings was optimised by FA spraying application relative to the Cd treatment, according to the results of ^13^C and ^15^N tracing.

**Conclusion:**

Conclusively, our results suggested that the inhibitory effect of Cd on apple seedlings root growth was alleviated by FA through regulating antioxdiant capacities and C and N metabolism.

## Introduction

Cadmium (Cd) has become a major concern worldwide, and pollution due to Cd is becoming increasingly severe due to the advances in anthropogenic activities (Duan et al., 2016; Argüello et al., 2019; Kaya et al., 2019);. Numerous studies have shown that excess Cd in plant environments reduces leaf chlorophyll biosynthesis, induces oxidative stress, and inhibits plant growth (Hasan et al., 2015; Luo et al., 2016; Podazza et al., 2016; Zhou et al., 2017). In addition, the Cd accumulated in agricultural products enters the food chain and poses a huge risk to human health (Zhuang et al., 2023). Apple, one of the most widely cultivated fruit in China, plays an important role in regional economic development and rural revitalization. Currently, due to sewage irrigation and chemical fertilizer and pesticide application, the problem of Cd pollution in orchards is becoming more and more prominent, seriously restricting apple plant growth, reducing fruit yields, and causing huge potential health threats to humans (He et al., 2020; Zhuang et al., 2023). Therefore, how to minimize the negative effects induced by Cd poisoning on the growth and development of apple plants through establishing reliable methods to enhance the Cd tolerance of apple plants is currently an urgent problem for the sustainable development of China’s apple industry.

In recent years, considerable research progress has been carried out to explore reliable methods to mitigate Cd-induced negative influence in plants including the exogenous application of materials such as plant growth regulators (Parvaiz et al., 2016; Wang et al., 2016; He et al., 2020). As a reactive, relatively low molecular weight humic acid substance, fulvic acid (FA) has been successful in alleviating the adverse effects induced by external abiotic stresses (Suh et al., 2014; Canellas et al., 2015; Wang et al., 2019; Chen et al., 2022)—for instance, the treatment of FA protected the soybean from heat and salt stresses (Dinler et al., 2016). By increasing the proline levels, FA application in maize reduced the negative effects of drought stress (Anjum et al., 2011). The application of FA also protected *Brassica napus* from water stress by modulating the antioxidant enzyme activities (Lotfi et al., 2015). In wheat, FA application reduced chromium (Cr) toxicity by regulating the photosynthetic pigments (Ali et al., 2015). However, little is known about the mechanisms for using FA to increase apple trees’ resistance to Cd toxicity and to mitigate the inhibitory effects of Cd stress on apple plants’ growth.

The process of plant growth is the accumulation of plant biomass. As the two basic and metabolic processes in higher plants, how plants grow and develop as well as plant stress tolerance promotion are highly dependent on the close relationship between C and N metabolism (Han et al., 2016; Ren et al., 2020; Liu et al., 2022). Therefore, it is very important to maintain C and N assimilation homeostasis to mitigate the inhibitory effects of external stress conditions on plant growth (Ren et al., 2021). Although numerous studies have shown that FA application could promote plant growth through the regulation of C and N metabolism (Gao et al., 2022; Yu et al., 2023a), details regarding the effects of Cd stress on C and N metabolism in apple plants and whether FA is sufficient to mitigate the inhibitory effects of Cd stress on apple plants growth, from the perspective of the regulation of C and N metabolism, both remain unclear.

Here we used M9T337 (an apple rootstock) seedlings as experiment materials; the effects of FA addition or not on plant growth, photosynthetic indexes, antioxidant enzyme activities, and C and N metabolism-related enzyme activities of M9T337 under Cd stress were investigated. Simultaneously, we used isotopic (^13^C and ^15^N) labeling to study the effects of Cd or FA addition on C and N partitioning in apple seedlings. This study’s findings provides a theoretical reference regarding FA alleviation in Cd-induced plant growth inhibition in apple seedlings and promotes the application of FA in apple orchards with excessive soil Cd levels.

## Materials and methods

### Plant material and growth conditions

The M9T337 seedlings were incubated in a greenhouse at Shandong Institute of Pomology under natural light at 22–26°C and 7–10°C day and night temperatures and 60%–65% relative humidity. The seedlings with uniform height were selected and transplanted into 40 cm × 30 cm × 15 cm plastic basins containing 6 L of half-strength Hoagland’s nutrient solution (Hoagland and Arnon, 1950). Moreover, eight seedlings were maintained per basin in this experiment.

### Experimental design and sampling

A hydroponic experiment was carried out by growing the seedlings under four treatments as follows: nutrient solution alone (CK), nutrient solution containing Cd (Cd), nutrient solution along with FA sprayed on the leaves (CK+FA), and nutrient solution containing Cd and FA was sprayed on the leaves (Cd+FA). Here CdCl_2_·2.5 H_2_O was used as the only Cd source, and the concentration of the Cd treatment was 30 μM/L. The nutrient solution was changed every 3 days. FA purchased from Bio Aladdin (Shanghai, China) was sprayed on the leaves at every change of nutrient solution. The concentration of FA was 0.2 g/L FA. Plants (seedlings) were harvested after treatment for 15 days (the nutrient solution was changed five times in total). Each treatment contained 36 plastic basins, with eight seedlings in each basin and 12 basins pooled as a repeat, and each treatment was replicated three times. For the purpose of isotope labeling as well as excluding the influence of different treatments on the ^15^N and ^13^C natural abundances in seedling organs, each repeat was divided into two groups: one group was used for ^15^N and ^13^C labeling as well as the measurement of ^15^N and ^13^C abundances (marker group), and the other group (unlabeled group) was used for measuring other indices.

### Growth performance indexes

In this study, for each of the normal (unlabeled) groups of each treatment, three seedlings were selected at random for the root morphology measurements, and root total length (RL) and root surface area (RSA) were selected to evaluate the root morphology. After completing the root sample preparations as described by Xing et al. (2021), the values of RL and RSA were measured using the WinRHIZO software (Regent Instruments Canada, Inc.). The samples were prepared according to Xu et al. (2020). Subsequently, a 1/1,000 electronic balance was used to measure the dry weight. Meanwhile, the determination of root activity was also measured using the triphenyltetrazolium chloride (TTC) method of Chen et al. (2018).

### Photosynthetic parameter

Following the details presented by Xu et al. (2020), a LI-6400XT portable photosynthesis system (LI-COR, Lincoln, NE, USA) was employed to determine the *P*_n_ (net photosynthetic rate) and *G*_s_ (stomatal conductance). Moreover, a pulse-modulated chlorophyll fluorescence meter (PAM 2500, Walz, Germany) was used to analyze the chlorophyll fluorescence parameters.

### H_2_O_2_ and MDA levels

The hydrogen peroxide (H_2_O_2_) content was measured following the method of He et al. (2011). The malondialdehyde (MDA) content was measured as described by Lei et al. (2007).

### Enzyme activities

In addition to leaf Rubisco (ribulose-1,5-biphosphate carboxylase-oxygenase) measurement based on the method of Hu et al. (2016a), the activities of nitrate reductase (NR), glutamine synthetase (GS), and glutamate synthase (GOGAT) both in roots and leaves were measured using the method presented by Hu et al. (2016b). The method reported by Rufly and Huber (1983) was adopted in this experiment to analyze the activity of sorbitol dehydrogenase (SDH). The activities of fructokinase (FRK) and hexokinase (HK) were measured according to the method of Li et al. (2012). Moreover, the activity levels of SOD, CAT, and POD were analyzed according to He et al. (2011).

### ^13^C and ^15^N isotope labeling

The basins of the marker group in each treatment were used for ^15^N and ^13^C labeling. For ^15^N isotope tracing, 0.5 g Ca(^15^NO_3_)_2_ from Shanghai Chemical Research Institute, China, was added to the nutrient solution at each change of the solution. The total amount of Ca(^15^NO_3_)_2_ used in each basin was 2.5 g. After 12 days of treatment, the ^13^C isotope tracing was initiated, following the method of Xu et al. (2020). In brief, the basins of the marker group in each treatment, markers (Ba^13^CO_3_, 98% independence), fans, and reduced iron powder were placed into a sealed marking room. Each basin corresponded to one marking room, and the dosage of each basin was 2 g. The labeling work lasted for 4 h. Hydrochloric acid was injected into the beaker with a syringe every 0.5 h in order to maintain the concentration of ^13^CO_2_. At the same time, three other plants (seedlings) from the unlabeled group were selected to measure the ^13^C natural abundance. Similar with the unlabeled group, samples of the marker group in each treatment were harvested at the end of the experiment (after 15 days of treatment).

Each plant sample was divided into roots, stems, and leaves and used to measure the ^15^N and ^13^C abundance, following the method of Xu et al. (2020). A MAT-251-Stable isotope ratio mass spectrometer was used to measure ^15^N abundance, while a DELTAVplusXP advantage isotope ratio mass spectrometer was selected to determine ^13^C abundance. Finally, the ^15^N- and ^13^C-related indexes were calculated using the following equations:

Calculation of ^15^N


(1) Ndff (%) =abundance ofN15 in plant-natural abundance ofN15abundance ofN15 in fertilizer-natural abundance ofN15× 100%

The Ndff (%) in Equation (1) Ndff refers to the contribution rate of ^15^N absorbed from fertilizer and distributed by plant organs relative to the total nitrogen of plant organs and reflects the ability of plant organs to absorb and regulate ^15^N fertilizer.


(2) N15 absorbed by each organ=Ndff(%)×total N content (mg)

The total N content (mg) in Equation (2) refers to organ total N content (mg).


(3) N15 use efficiency (NUE15)=the totalN15 absorption amount divided by the totalN15  application amount

In Equation (3), the total ^15^N absorption refers to the total amount obtained by adding the ^15^N of each organ.

Calculation of ^13^C


(4) Abundance ofC13:Fi(%)=(δC13+1000)×RPBD(δC13+1000)×RPBD+1000× 100%

In Equation (4), the value of R_PBD_ (standard ratio of carbon isotope) is set to 0.0112372.


(5) Content ofC13 in each organ:C13i(mg)=Ci×(Fi−Fnl)100× 1,000

In Equation (5), the value of C_i_ (C content of each organ) is the product of the organ’s dry matter (g) and the organ’s total carbon content (%); F_nl_ represents the ^13^C natural abundance of each organ.


(6) C13 partitioning rate:C13(%)=C13iC13net absorption×100%

In Equation (6), ^13^C_net absorption_ refers to the total amount obtained by adding the ^13^C of each organ.

### RNA isolation and qRT-PCR analysis

Total RNA was extracted from 0.1 g of the roots using the RNAiso Plus extraction kit (Takara, Otsu, Shiga, Japan). Subsequently, RNA extraction, reverse transcription, and qRT-PCR procedures followed a previously reported procedure (Yu et al., 2023a). The relative gene expression levels were normalized according to the 2^–ΔΔCT^ method, and the *MdActin* gene was used as the internal reference gene. Three biological replicates per treatment and three technical replicates per sample were used in the assay. The primers are listed in **Supplementary Table S1**.

### Statistical analysis

This experiment was conducted using Microsoft Excel for data collection and SPSS 21.0 (SPSS, Inc., Chicago, IL, USA) for data analysis using one-way analysis of variance (ANOVA) and a *post hoc* test (Duncan’s). The differences were considered statistically significant at a probability level of *P*< 0.05.

## Results

### Plant growth

**Figure 1:**
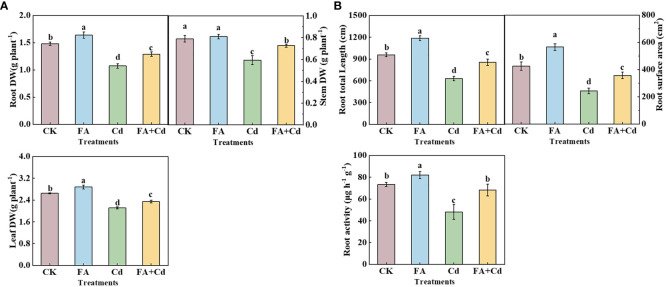
Organs’ dry matter weight **(A)**, root total length, surface area of seedlings as well as root activities **(B)** under different treatments. CK, control—nutrient solution alone; FA, nutrient solution with FA; Cd, nutrient solution containing Cd; Cd+FA, nutrient solution containing Cd and the spraying application of FA. The vertical bars on the histograms indicate ± SD. The different letters indicate statistically significant differences (*P*< 0.05).

Compared with CK, Cd addition alone (Cd treatment) resulted in a significant decrease in the dry weight of the seedlings’ organ (**Figure 1A**). In contrast, the Cd-induced decrease was significantly decreased by FA spraying application under Cd exposure condition relative to the Cd treatment. Consistent with Cd-induced root dry weight inhibition, the seedlings’ RL (root total length) and RSA (root surface area) were both significantly decreased under Cd addition (Cd and Cd+FA) conditions relative to CK. In contrast, the RL and RSA were both optimized by FA spraying application under Cd stress, which were 35.55% (RL) and 46.78% (RSA), respectively, higher than that treated by the Cd treatment. Moreover, the Cd treatment significantly lowered the seedling root activities, and FA spraying application could alleviate the Cd-induced adverse effect on root activity, which was 53.52% higher than that of the Cd treatment (**Figure 1B**).

### H_2_O_2_, and MDA levels and antioxidant enzyme activity

**Figure 2:**
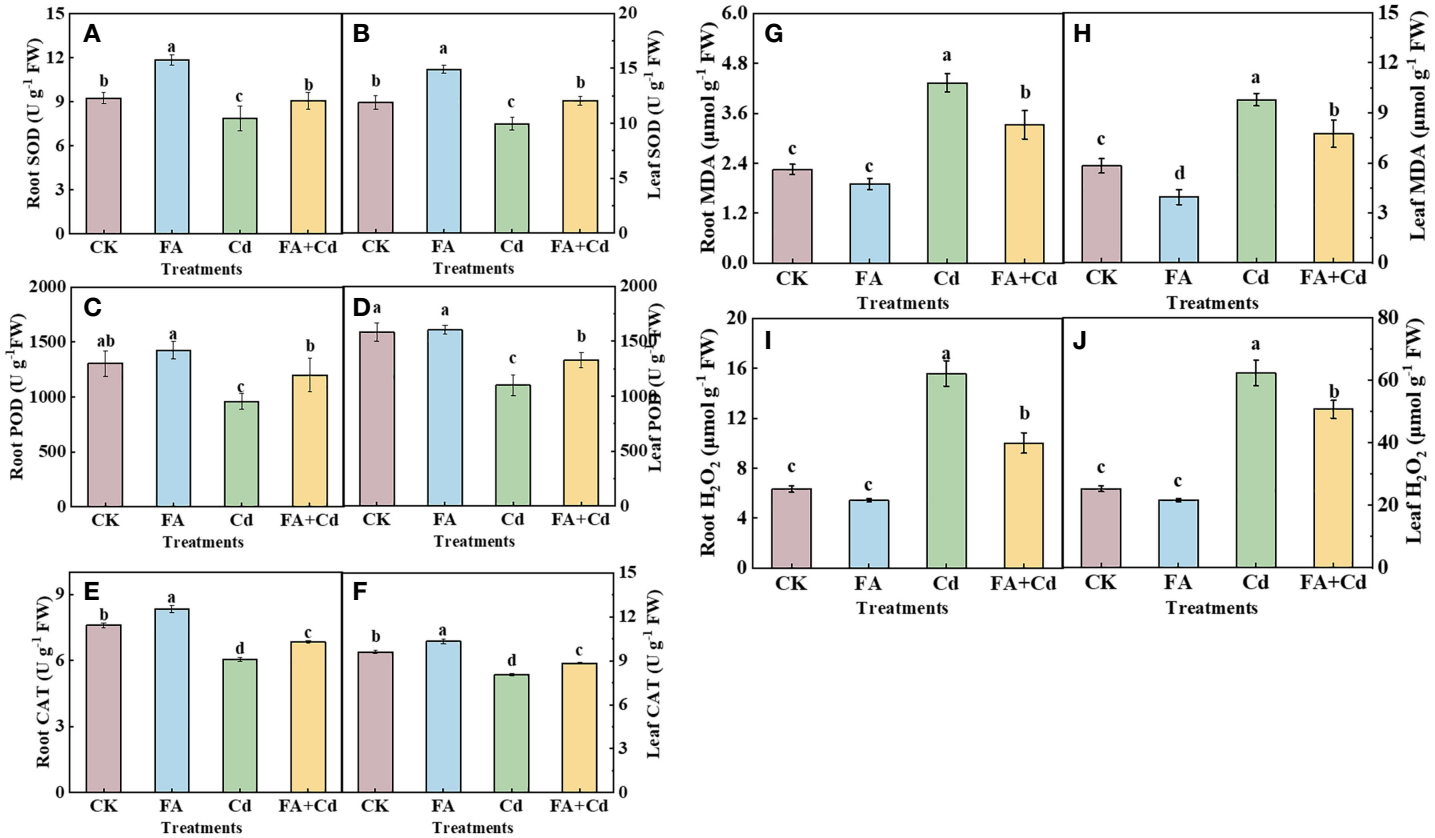
SOD, POD, and CAT activities in roots **(A, C, E)** and leaves **(B, D, F)** as well as MDA and H_2_O_2_ contents in roots **(G, H)** and leaves **(I, J)**. The different letters indicate statistically significant differences (P< 0.05).

Irrespective of FA spraying, the addition of Cd (Cd and Cd+FA) treatments both resulted in higher H_2_O_2_ and MDA levels in roots than that of CK (**Figures 2G-J**). The highest H_2_O_2_ and MDA levels were observed under the Cd treatment, while the lowest were under CK. However, no significant decrease was observed in root H_2_O_2_ and MDA levels between the FA treatment and CK. Relative to CK, the Cd treatment largely elevated the contents of H_2_O_2_ and MDA in leaves, which were 2.46 (H_2_O_2_) and 1.68 (MDA) times that of CK, respectively. However, under Cd stress condition, the increases of the leaves’ H_2_O_2_ and MDA levels induced by Cd stress were weakened by FA spraying application, which were decreased by 35.83% and 23.33%, respectively, compared to that in the Cd treatment.

Subsequently, we determined the SOD, POD, and CAT activities in leaves and roots, respectively, and observed that the activities of enzymes mentioned above were both decreased under Cd exposure condition relative to CK (**Figures 2A-F**). In contrast, a promoting effect was found under the FA treatment compared to that of CK, showing higher SOD and CAT activities in roots and SOD activity in leaves than that of CK. Meanwhile, the Cd-induced decreases of enzyme activities were both significantly weakened when FA was sprayed on the Cd-treated seedlings’ leaves.

### Photosynthetic characteristics

**Figure 3:**
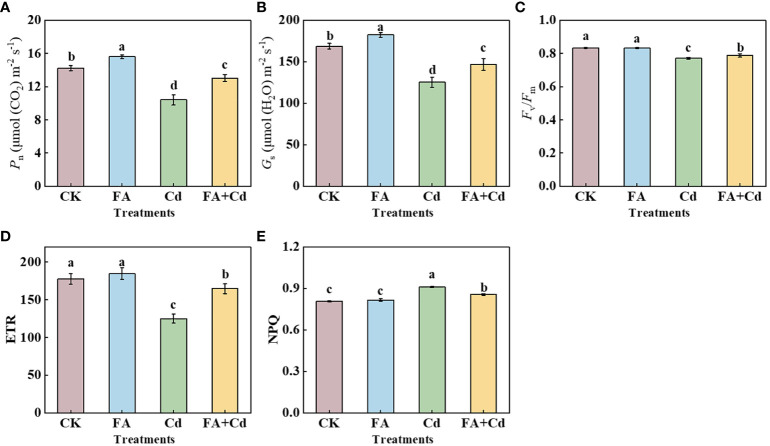
*P*_n_ **(A)**, *G*_s_ **(B)**, *F*_v_/*F*_m_ **(C)**, ETR **(D)**, and NPQ **(E)** in leaves under different treatments. CK, control—nutrient solution alone; FA, nutrient solution with FA; Cd, nutrient solution containing Cd; Cd+FA, nutrient solution containing Cd and the spraying application of FA. The vertical bars on the histograms indicate ± SD. The different letters indicate statistically significant differences (*P*< 0.05).

Under conditions without Cd, FA spraying application increased the leaf *P*_n_ and *G*_s_ by 9.63% (*P*_n_) and 8.10% (*G*_s_) compared to CK (**Figures 3A, B**). However, 15 days of exposure to Cd stress resulted in an apparent decrease in *P*_n_ and *G*_s_ compared to CK. This Cd-induced reduction in *P*_n_ and *G*_s_ was alleviated by FA treatment. The *P*_n_ and *G*_s_ values under Cd+FA were 25.29% and 17.03% higher than that under Cd alone. Furthermore, Cd significantly decreased the *F*_v_/*F*_m_ value compared to CK (0.92 times that of CK), while FA spraying application mitigated the Cd-induced inhibition of *F*_v_/*F*_m_ (**Figure 3C**). Different from the changes in the *F*_v_/*F*_m_ among the CK, Cd, and Cd+FA, no significant difference was observed between the CK and the FA treatments. The Cd+FA treatment also elevated the ETR in seedlings compared with the Cd treatment (Cd addition alone); the ETR under the Cd+FA treatment was 31.74% higher than that under the Cd treatment. However, NPQ showed an opposite trend in the apple seedlings. The highest NPQ was detected in the seedlings under Cd treatment (Cd), while the application of FA spraying under Cd stress decreased NPQ compared to Cd alone (**Figure 3E**).

### C metabolism-related enzymes

**Figure 4:**
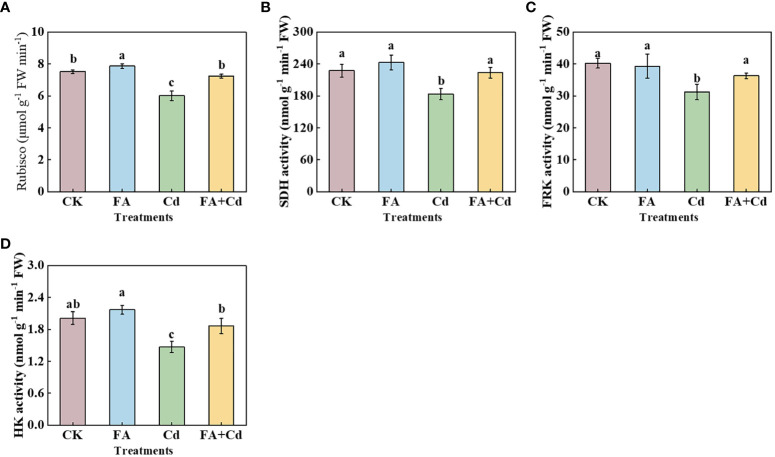
Rubisco activity in leaves **(A)** and SDH, FRK, and HK activities in roots **(B–D)** under different treatments. CK, control—nutrient solution alone; FA, nutrient solution with FA; Cd, nutrient solution containing Cd; Cd+FA, nutrient solution containing Cd and the spraying application of FA. The vertical bars on the histograms indicate ± SD. The different letters indicate statistically significant differences (*P*< 0.05).

Among all the treatments, the leaves’ Rubisco activity was highest in the FA treatment, and the lowest was obtained under the Cd treatment. Compared with the Cd treatment, the Rubisco activity of leaves was elevated by 20.50% in the Cd+FA treatment (**Figure 4A**). Moreover, under Cd stress condition, FA spraying application weakened the Cd-induced inhibition in the activity of these enzymes in roots (**Figures 4B-D**), which were elevated by 22.00% (SDH), 26.53% (HK), and 16.25% (FRK), respectively, compared to the Cd treatment.

### ^13^C accumulation, distribution, and transportation

**Figure 5:**
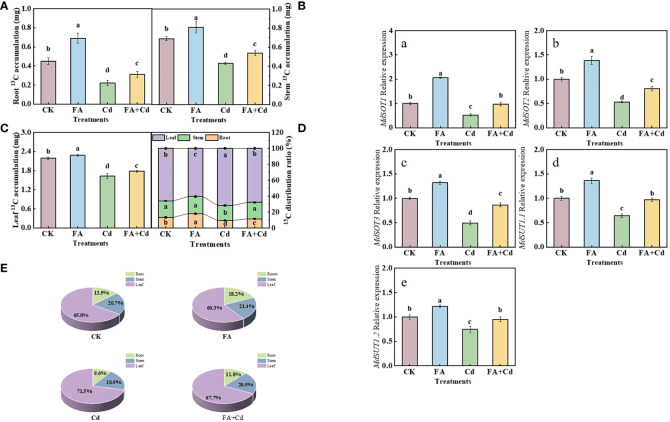
^13^C accumulation of seedlings **(A–C)**, organs’ ^13^C distribution rate **(D, E)** as well as roots’ *MdSOTs* and *MdSUTs* gene expression **(A–E)** under different treatments. CK, control—nutrient solution alone; FA, nutrient solution with FA; Cd, nutrient solution containing Cd; Cd+FA, nutrient solution containing Cd and the spraying application of FA. The vertical bars on the histograms indicate ± SD. The different letters indicate statistically significant differences (*P*< 0.05).

The results of ^13^C labeling indicated that Cd addition and FA spraying applications significantly influenced the ^13^C accumulation and ^13^C distribution rate in each organ. As shown in **Figures 5A–C**, compared with CK, the application of FA spraying significantly elevated the ^13^C accumulation in seedlings. In contrast, under stress conditions, compared with FA absence treatment (Cd addition alone), the ^13^C accumulation in seedlings was significantly elevated when FA was sprayed, which was increased by 40.91% (root), 25.58% (stem), and 9.20% (leaf), respectively, compared to that in the Cd treatment. Subsequently, we further observed that, regardless of the treatment of the study, the highest ^13^C distribution rate was detected in the leaves, followed by the stem, and the lowest in the roots. The Cd treatment resulted in the lowest ^13^C distribution rate in roots and the highest in leaves, while an opposite trend was observed under FA treatment. Besides this, treating apple seedlings under Cd stress with FA significantly increased the ^13^C distribution rate in roots and decreased that in leaves compared with the Cd treatment, which was 22.22% (^13^C distribution rate in roots) higher and 5.30% (^13^C distribution rate in leaves) lower, respectively, than that of the Cd treatment (**Figures 5D, E**).

We measured three *MdSOTs* (*MdSOT1*, *MdSOT2*, and *MdSOT3*) and two *MdSUTs* (*MdSUT1.1* and *MdSUT1.2*) expression levels in this study and observed that these five genes’ expression levels were all upregulated by FA spraying application under non-Cd addition condition, relative to CK (**Figure 5A-E**). Meanwhile, Cd stress downregulated the expression of these genes. However, FA spraying application alleviated this reduction in gene expression under Cd exposure.

### N metabolism-related enzymes

**Figure 6:**
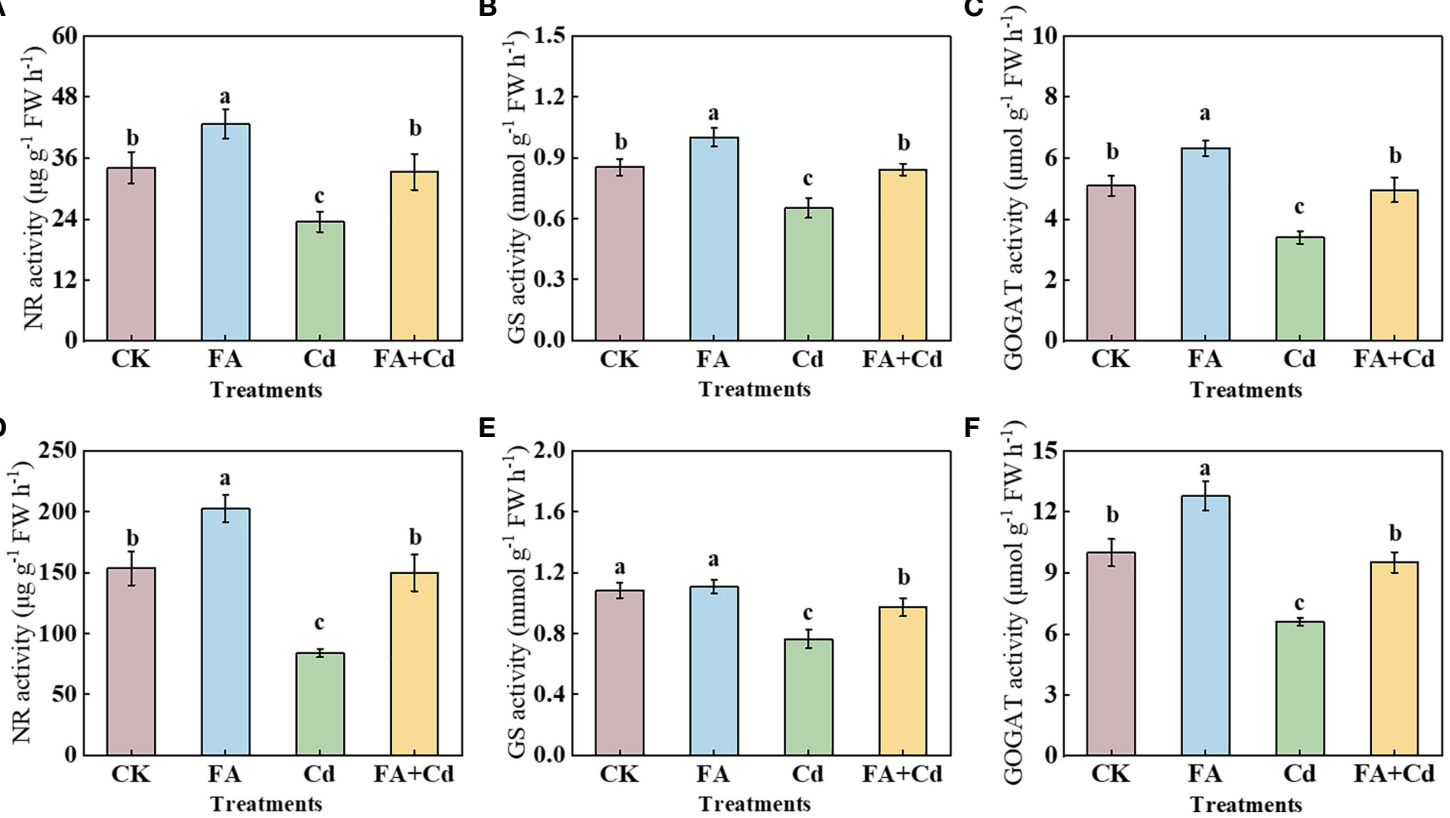
Activities of NR, GS, and GOGAT in leaves **(D–F)** and roots **(A–C)** under different treatments. CK, control—nutrient solution alone; FA, nutrient solution with FA; Cd, nutrient solution containing Cd; Cd+FA, nutrient solution containing Cd and the spraying application of FA. The vertical bars on the histograms indicate ± SD. The different letters indicate statistically significant differences (*P*< 0.05).

Compared with CK, the NR activities in leaves and roots, the GS activity in roots, and the GOGAT activities in the leaves and roots of FA-treated-apple seedlings were 32.00%, 25.24%, 17.65%, 28.03%, and 24.36% higher than those under CK. The Cd treatment also significantly influenced the activity of these enzymes. Compared with CK, Cd stress reduced the activities of NR, GS, and GOGAT in leaves and roots. However, FA spraying application alleviated the negative effect of Cd on the enzymes (**Figures 6A-F**).

### NRT gene expressions in roots

**Figure 7:**
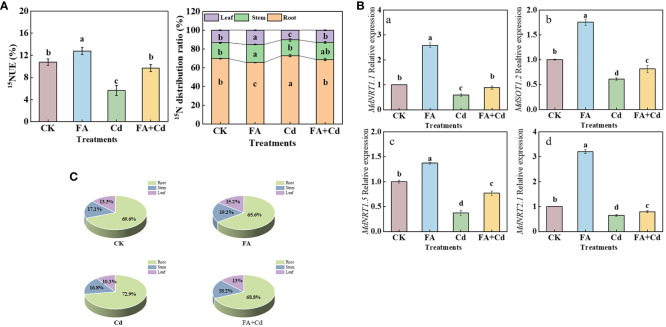
^15^NUE of seedlings **(A)**, organs’ ^15^N distribution rate **(B, C)**, and the *MdNRTs* gene expression (a–e) in roots under different treatments. CK, control—nutrient solution alone; FA, nutrient solution with FA; Cd, nutrient solution containing Cd; Cd+FA, nutrient solution containing Cd and the spraying application of FA. The vertical bars on the histograms indicate ± SD. The different letters indicate statistically significant differences (*P*< 0.05).

Four *MdNRTs* (*MdNRT1.1*, *MdNRT1.2*, *MdNRT1.5*, and *MdNRT2.1*) gene expression levels were analyzed in this study. Compared with CK, the FA treatment upregulated the expression of all these genes, in the apple seedlings, while the Cd treatment downregulated their expression (**Figures 7A-D**). Moreover, although the expression levels of these genes under the Cd+FA treatment were higher than those under the Cd treatment, their expression levels were still lower than that of CK.

### ^15^N accumulation and ^15^N distribution ratio

As shown in **Figure 7A**, the FA treatment resulted in the highest ^15^NUE value (1.18 times that of CK), while the Cd treatment resulted in the lowest (0.52 times that of CK). Compared with the Cd treatment, ^15^NUE value was increased by the Cd+FA treatment, which was 71.38% higher than that of Cd treatment. Subsequently, we measured the organs’ ^15^N distribution ratio and observed that, under Cd treatment, the ^15^N distribution ratio in leaves was significantly lower than that of CK, which was 0.78 times that of CK. However, FA spraying application under Cd exposure condition significantly optimized the ^15^N distribution ratio in leaves and roots. Compared with the Cd treatment, the Cd+FA treatment significantly increased the ^15^N distribution ratio in leaves (26.09% higher than that of Cd treatment) and decreased that in roots (**Figures 7B, C**).

## Discussion

### FA spraying application improves the antioxidant system in apple plants under Cd stress

Dry weight is an important feature that reflects the growth characteristics of plants. Researchers analyze this crucial feature to assess the influence of different treatments on the growth of seedlings. The present study found that Cd stress decreased the dry weight of roots and all aboveground parts of the apple seedlings, indicating suppressed seedling growth under Cd stress, which is consistent with the findings of Zhou et al. (2016, 2017). In contrast, the negative effect caused by Cd stress was significantly reduced by FA spraying application. Seedlings treated with the Cd+FA treatment presented a higher dry weight than that of the Cd treatment (Cd addition without FA application). These results were similar with those of Wang et al. (2019) in lettuce. The root system provides support for the body of the plant, and the water and nutrients required for the process of plant growth are taken up from the external environment by the root system. Moreover, roots are, in most cases, the first to be exposed to abiotic stressors in the external environment, and the response of roots (the key organ system absorbing nutrients) to changes in the external environment could be the initial driving force for influencing plant growth (Vadez, 2014; Shi et al., 2023). Therefore, improving the growth and development of root under conditions of stress may effectively enhance the tolerance of apple plants. We further analyzed the effect of Cd stress and FA application on the root morphology parameters of apple seedlings. Similar to the changes in root dry weight, the RL and RSA of apple seedlings decreased after exposure to Cd stress (**Figure 1B**), indicating that root growth was inhibited by Cd stress. Previous studies have pointed out that the production and scavenging of reactive oxygen species (ROS) occur in a relatively stable state (El-Shabrawi et al., 2010). The present study also found that the levels of MDA and H_2_O_2_ in roots under Cd stress were obviously higher than that of CK, indicating that Cd stress (the abiotic stressor in this study) increased the ROS production. Besides this, the Cd treatment also decreased the activities of SOD, POD, and CAT in roots, indicating a decrease in ROS scavenging ability and an imbalance in ROS in the roots of the seedlings under Cd stress. However, FA spraying application decreased the levels of MDA and H_2_O_2_ but increased the activities of SOD, POD, and CAT in apple seedlings under Cd stress (**Figure 2**), indicating that FA weakened the Cd-induced root growth inhibition by enhancing the scavenging of ROS and maintaining a balance in ROS production and scavenging. Moreover, FA downregulates the gene expression of transporter proteins and reduces Cd uptake under Cd stress, which could be another important reason to explain the response of root growth and development to Cd stress and FA application (Chen et al., 2022).

### FA spraying application enhances C metabolism in apple seedlings under Cd stress condition

As the basic life activity of plants, previous studies had already pointed out that Cd stress could inhibit leaf photosynthesis (He et al., 2020). In this study, the *P*_n_ and *G*_s_ values of leaves decreased under Cd stress (**Figures 3A, B**). In contrast, these inhibitions were both reduced when FA was applied in a Cd stress environment, which was consistent with the study of Chen et al. (2022). The *G*_s_ under the Cd+FA treatment was higher than that under the Cd treatment, indicating that the leaf CO_2_ absorption ability could be elevated by FA application in the Cd stress condition to a certain degree. These results may be beneficial to explain the difference in seedling total ^13^C accumulation treated with Cd or FA spraying (**Figure 5A**). In the present study, we observed that Cd stress significantly decreased the value of *ETR*, which was consistent with the findings obtained by Gong et al. (2017), who reported that one of the sites for Cd destruction was the electron transport chain. In addition, the study detected that the *ETR* was elevated under the Cd+FA treatment compared to the Cd treatment (**Figures 3C-E**), indicating that the damage Cd stress caused to the electron transfer chain was mitigated by FA spraying application. These benefits (the stability of the photosynthetic apparatus and the efficient transport of electron transfer) caused by FA application under Cd stress condition could provide a basis (a stable reducing force) for the enhancement of photosynthesis and photosynthesis media C (carbon) assimilation.

The CO_2_ (C) assimilation and the transport of photoassimilates are the basis of plant organ growth (Merlo and Passera, 1991; Lambers et al., 2002; Sha et al., 2020; Xu et al., 2023). Detailed analysis revealed that Cd treatment reduced the activity of Rubisco, indicating that the C assimilation could be somewhat inhibited under Cd stress condition. These results might be favorable to analyze why seedlings treated with the Cd treatment had a lower ^13^C accumulation than that of CK. According to the results presented by ^13^C labeling, we found that ^13^C accumulation of seedlings under the Cd treatment was obviously lower than that of CK. In contrast, this reduction was significantly decreased by FA spraying application under Cd stress condition (**Figures 5A-C**). The reason might be related with the elevation of Rubisco activity and light energy absorption and harvesting under the Cd+FA treatment (**Figure 4A**). The transport of photoassimilates has a vital role on root system construction and the ability of nutrient absorption by the root system (Xu et al., 2020; Yu et al., 2023b). Lyu et al. (2023) observed that the inhibition of the transport of photoassimilates and the weakness of leaf C assimilation caused by stress conditions could negatively influence the photosynthetic electron transport chain and result in an elevation of ROS levels. In this study, we found that the Cd treatment resulted in the highest leaf ^13^C distribution rate, the lowest root ^13^C distribution rate as well as the lowest seedling ^13^C accumulation (**Figures 5D, E**). These results could somewhat help to explain why seedlings treated with the Cd treatment had a higher H_2_O_2_ level in leaves and poor photosynthetic performance than other treatments. However, compared with the Cd treatment, the Cd+FA treatment significantly increased the seedlings’ root ^13^C accumulation. These observations collectively suggest that Cd stress inhibited the leaf-to-root translocation of photoassimilates, while FA spraying application alleviated this inhibition, which could be conducive to explain why seedlings treated with the Cd+FA treatment had a higher root dry weight than those of the Cd treatment to a certain degree (**Figure 2B**). *MdSOTs* and *MdSUTs* are extensively involved in the translocation of photoassimilates (Zhao et al., 2020). We observed that the root *MdSOT1*, *MdSOT2*, *MdSOT3*, *MdSUT1.1* and *MdSUT1.2* gene expressions were downregulated by Cd addition relative to the CK (**Figures 5A-E**). Moreover, we noticed that the roots’ SDH, FRK, and HK activities were also decreased by the Cd treatment. In contrast, these reductions were weakened by FA spraying application under Cd stress condition (**Figures 4B-D**). Combined with relevant research progress obtained in this study, we summarized as follows: Firstly, FA spraying application under Cd stress obviously optimized the leaf photosynthetic performance and reduced the negative influence on leaf C assimilation caused by Cd stress and enhanced the leaf photosynthetic product synthesis capacity, thus elevating source strength. Secondly, root sugar metabolism enzyme activities were elevated by the Cd+FA treatment relative to the Cd treatment, indicating that the process of root sugar metabolism was enhanced, which not only provides energy for root growth but also enhances the competitiveness of the root for photosynthetic products and promoting the translocation of photoassimilates to a certain extent. Finally, the upregulation of root sugar transport-related gene expression under the Cd+FA treatment could also be conducive to explain the enhancement of the leaf to root photoassimilate transport. In conclusion, the role of FA in promoting seedling root growth under Cd stress condition can be expressed through the optimization of leaf photosynthesis, root C metabolism process as well as the distribution of photoassimilates.

### FA spraying application enhances N metabolism in apple seedling under Cd stress condition

N, an essential macro-nutrient, is closely related to various physiological and metabolic activities of plants, such as organ construction and leaf photosynthesis (Titus and Kang, 1982; Warner et al., 2004; Tavares et al., 2019; Xu et al., 2020). Improving the absorption and utilization of N by plants promotes growth and enhances stress tolerance under unfavorable conditions (Yuan et al., 2007). In this study, the results of ^15^N labeling showed that, compared with CK, the Cd treatment obviously decreased the seedling ^15^NUE value (**Figure 7A**), indicating that the seedlings’ N absorption and utilization were obviously inhibited under Cd stress condition. However, this inhibition induced by Cd stress was somewhat weakened by FA spraying application. One of the reasons might be due to the optimization of root morphology (RL and RSA) and the elevation of root activity by FA addition under Cd exposure condition (**Figure 1B**), which improved the absorption of N in nutrient solution by roots. The uptake of N by plants is an energy-consuming process (Bloom, 2015). In this study, we observed that the accumulation of ^13^C-photoassimilates in roots and sugar metabolism-related enzyme activities was enhanced by FA spraying application under Cd stress condition (**Figures 4B–D**, **5A**), suggesting that the spraying of FA under Cd exposure condition can provide sufficient energy to support N uptake in the root system of apple seedlings by promoting the transport of photosynthetic products to the root system as well as increasing the intensity of sugar metabolism in the root system. Previous studies have reported that external environmental conditions could obviously influence the root *NRT* gene expression (Xing et al., 2021, 2022). Moreover, Tian et al. (2023) pointed out that the *NRT* gene expression level in root could determine the strength of nitrate uptake to a certain degree. Thus, changes in root *NRT* gene expression level in response to Cd exposure or FA spraying application may somewhat explain why FA-treated seedlings have higher ^15^NUE values than non FA-treated seedlings under Cd stress condition. In this study, under normal condition, compared with CK, FA spraying application promoted the expression of *MdNRT1.1*, *MdNRT1.2*, *MdNRT1.5*, and *MdNRT2.1*. Moreover, we observed that these genes’ expressions in roots were obviously downregulated under the Cd treatment relative to CK. In contrast, compared with the Cd treatment, the Cd+FA treatment significantly promoted the expression of *MdNRT1.1*, *MdNRT1.2*, *MdNRT1.5*, and *MdNRT2.1* (**Figures 7A-D**), indicating that the strength of nitrate (the only N source in this experiment) absorption was promoted. Earlier studies performed by Xu et al. (2020, 2022) both observed that the accumulation of potassium (K) could be favorable to promote the upregulation of *NRT* gene expression in roots. Moreover, Bayat et al. (2021) observed that FA application could promote the absorption of K in yarrow (*Achillea millefolium* L). Therefore, the change of root *NRT* gene expression level between the Cd and the Cd+FA treatments might be related with the improvement of K absorption by plants under FA spraying application.

After being absorbed by roots, nitrate (NO_3_^−^) is assimilated through a range of N metabolism enzymes (Xu et al., 2012). As the key enzymes widely participate in the plants’ N metabolism process, the activities of NR, GS, and GOGAT could somewhat reflect the efficiency of plant N metabolism (Armengaud et al., 2009; Wen et al., 2019; Gao et al., 2022). We observed that, under stress condition, compared with CK, Cd stress treated-seedlings had lower NR, GS, and GOGAT activities in roots and leaves (**Figure 6**). These results indicated that Cd poisoning could reduce the plant N assimilation process. A further analysis showed that, compared with the Cd treatment, the Cd+FA treatment significantly minimized the negative influence on NR, GS, and GOGAT activities (**Figure 6**). The elevation of the leaves’ GS and GOGAT activities under the Cd+FA treatment might dissipate excess energy in leaves and reduce photodamage in chloroplasts, which might be favorable to explain why FA-treated seedlings under Cd stress condition had a better photosynthetic performance than that of Cd treatment. The root system is a typical non-photosynthetic tissue and the dominant site for taking up N from the plant. Therefore, the above-mentioned changes in the accumulation of ^13^C-photosynthetic products and the activities of enzymes involved in root sugar metabolism could, to some extent, explain the differences in the activities of root N metabolism enzymes among various treatments.

N is a massive mineral element that is closely linked to the photosynthesis of the apple plant (Xu et al., 2020). Leaves are the main site of photosynthesis in plants. The enzymes and photosynthetic pigments required for photosynthesis are provided by N metabolism (Reguera et al., 2013). Scholars such as Xu et al. (2022) and Yu et al. (2023b) both pointed out that the alleviation of stress-induced root growth inhibition was closely related to the strength of the root-to-leaf transport of N in plant. The ^15^N labeling results showed that the lowest leaf ^15^N distribution ratio was observed under the Cd treatment (**Figures 7B, C**), indicating that the bottom (root) to top (leaf) transport of N was significantly inhibited. These results could also somewhat explain why Cd stress-treated seedlings had a poor photosynthetic performance. In contrast, the inhibition caused by Cd poisoning was reduced by FA spraying application. Xu et al. (2022) observed a positive correlation between *NRT1.5* gene expression level and root-to-leaf translocation of N. Therefore, a higher *NRT1.5* gene expression level under the Cd+FA treatment could be conducive to explain why seedlings treated with Cd+FA had a higher leaf N distribution rate than that of the Cd-treated ones (**Figure 7D**).

## Conclusion

**Figure 8:**
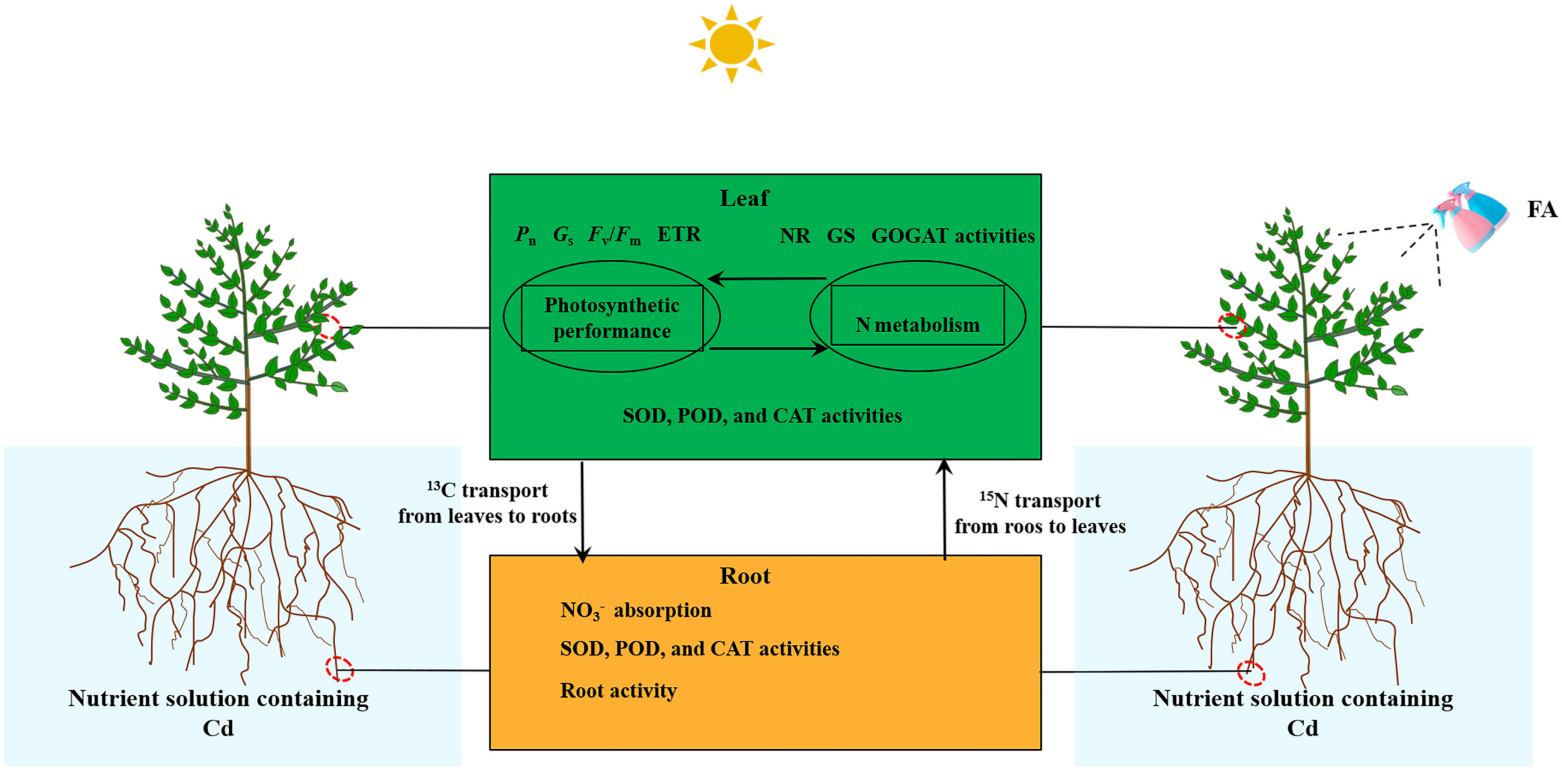
Schematic model displaying the role of FA-mediated alleviation in Cd-induced root growth inhibition in apple seedlings.

In conclusion, the FA alleviation in Cd-induced apple seedlings’ root growth inhibition was associated with the following characteristics (**Figure 8**): (i) the enhanced antioxidant enzyme activities in roots and leaves, (ii) the enhanced leaf photosynthesis as well as the elevated C and N metabolism-related enzyme activity, and (iii) the more rational distribution of C and N in seedlings. Overall, this study offers a fresh train of thought into the promotion of Cd stress-treated apple plants’ root growth caused by FA spraying application.

## Data availability statement

The original contributions presented in the study are included in the article/**Supplementary Files**, further inquiries can be directed to the corresponding author.

## Author contributions

BY: Methodology, Writing – original draft, Writing – review & editing, Data curation, Formal Analysis. XX: Methodology, Writing – review & editing. PN: Conceptualization, Writing – review & editing. NL: Writing – review & editing. LW: Funding acquisition, Methodology, Writing – original draft, Writing – review & editing.
