# Supplementary material for: Fulvic acid alleviates cadmium-induced root growth inhibition by regulating antioxidant enzyme activity and carbon–nitrogen metabolism in apple seedlings
Source: Front Plant Sci. 2024 Apr 2;15:1370637. doi: 10.3389/fpls.2024.1370637 (PMC11072189; doi:10.3389/fpls.2024.1370637)
Supplement: Supplementary file 1 [file Table_1.docx]

**Table S1** Primer sequences for PCR

| Gene | Forward primer sequence (5′→3′) | Reverse primer sequence (5′→3′) |
| --- | --- | --- |
| *MdACTIN* | TGGTGTCATGGTTGGTATGG | CCGTGCTCAATGGGATACTT |
| *MdNRT1.1* | TTGGCTCTGACCAGTTCGAC | ACGGTACTGTCTTGTGCCTG |
| *MdNRT1.2* | TGTTGACTGGAGAAGCCGAC | GTGACATCGTTTGCCGCTTT |
| *MdNRT1.5* | GTGGTCATGAAGTTCTCGACAGAG | TCAGACTTTCAAGTTTCCTTGATCAT |
| *MdNRT2.1* | TTGAGAGTTTGACAAGATAAACGCA | AACTGACGGGTGGGGAAATC |
| *MdSUT1.1* | GTGGTAATTTACCGGCATTTGTCG | AAGCTAGAGGCCGTAGGGCAAG |
| *MdSUT1.2* | CGAAGACAGAAAGTGTGTGTCCTG | GTAGGGGGTGAGGAGGGAGAG |
| *MdSOT1* | GACAGAACCTCAGACTCCAAAG | TGACCAGAGACCTGAACGATA |
| *MdSOT2* | CGTATCCAACTATGCCTTCTCC | GACACCGACGGCAAGAATAA |
| *MdSOT3* | GCCGGTACTCTAAACATCTACTC | GAAGATGACTCCCGCAAGAA |
